# Vection underwater illustrates the limitations of neutral buoyancy as a microgravity analog

**DOI:** 10.1038/s41526-023-00282-3

**Published:** 2023-06-10

**Authors:** Nils-Alexander Bury, Michael Jenkin, Robert S. Allison, Rainer Herpers, Laurence R. Harris

**Affiliations:** 1 Institute of Visual Computing, Hochschule Bonn-Rhein-Sieg, Grantham-Allee 20, 53757 St. Augustin, Germany; 2 Centre for Vision Research, York University, 4700 Keele St., Toronto, ON M3J 1P3 Canada; 3 Dept. of Psychology, York University, 4700 Keele St., Toronto, ON M3J 1P3 Canada; 4 Department of Electrical Engineering & Computer Science, York University, 4700 Keele St., Toronto, ON M3J 1P3 Canada; 5 Faculty of Computer Science, University of New Brunswick, Fredericton, Canada

**Keywords:** Human behaviour, Neuroscience

## Abstract

Neutral buoyancy has been used as an analog for microgravity from the earliest days of human spaceflight. Compared to other options on Earth, neutral buoyancy is relatively inexpensive and presents little danger to astronauts while simulating some aspects of microgravity. Neutral buoyancy removes somatosensory cues to the direction of gravity but leaves vestibular cues intact. Removal of both somatosensory and direction of gravity cues while floating in microgravity or using virtual reality to establish conflicts between them has been shown to affect the perception of distance traveled in response to visual motion (vection) and the perception of distance. Does removal of somatosensory cues alone by neutral buoyancy similarly impact these perceptions? During neutral buoyancy we found no significant difference in either perceived distance traveled nor perceived size relative to Earth-normal conditions. This contrasts with differences in linear vection reported between short- and long-duration microgravity and Earth-normal conditions. These results indicate that neutral buoyancy is not an effective analog for microgravity for these perceptual effects.

## Introduction

As far back as the Gemini program (1965–1966), astronauts have used both large and small water tanks to provide a neutral buoyancy analog for space missions^1–3^. In such tanks, astronauts in simulated space suits “float” in a neutrally buoyant environment. Today, most space agencies with an astronaut program utilize neutral buoyancy training as part of their astronaut training program and to rehearse tasks, particularly extravehicular activities, that will be performed in space^4^.

Neutral buoyancy tanks permit six-degrees-of-freedom motions to be practiced, and experiments to be performed with full-scale mock-ups of space hardware. However, as with other space analog environments on Earth, the neutral buoyancy environment is not a perfect analog for microgravity. Properly weighted objects may float in neutral buoyancy, but water is not a vacuum, and the resistance of the water column and the inertia of water and its impact on objects does not match the effects experienced in air in a space station or in vacuum during extravehicular microgravity. Neutral buoyancy impacts other aspects of human perception and performance which may or may not match either Earth-normal behavior or behavior experienced in the environment found in other space analogs including human centrifugation^5^, microgravity aircraft flight^6–10^ or long-duration bed rest^11^. For example, Glass et al.^12^ found that participants swayed less following long-duration water immersion compared to performance prior to exposure. They noted that proprioception^13^, somatosensation and vision^14^, possibly because human eyes evolved for viewing in air, are less informative of postural changes underwater and hypothesized that the sway changes may reflect a downweighting of these cues and a concomitant upweighting of vestibular input. Jarchow and Mast^15^ reported that immersion and neutral buoyancy resulted in a head upward bias in the subjective horizontal body posture compared to performance on land in 3 of their 4 participants. Underwater immersion appears to influence both balance and orientation with respect to gravity, two critical functions of graviception.

Does neutral buoyancy interfere with perceptual systems and are those influences similar to those found in microgravity? In microgravity, both somatosensory and vestibular cues to the direction of gravity are compromised but in neutral buoyancy the vestibular cue remains unaffected while only the somatosensory cue, normally provided by pressure at the support surface, is disabled^16–18^. Capitalizing on the similarities, neutral buoyancy in underwater immersion with specialized visual environments (an inverted room) has been proposed as a model of space motion sickness^19^. Water immersion allows for six degree of freedom movement under visual-vestibular conflict while producing many, but not all, aspects of the reduced somatosensory cues encountered in space. As well as orientation, integration of visual, vestibular and somatosensory information is important for effective self-motion. Here we consider whether lack of the somatosensory cue alone affects the perception of self-motion (vection) or spatial distance under neutral buoyancy conditions.

Humans and other animals exploit a range of different cues to judge the distance that they have traveled. Visual, vestibular, efferent copy and a range of proprioceptive cues go into constructing this estimate^20^. Vestibular, visual, somatosensory and proprioceptive signals are combined at the earliest stages of vestibular processing such as the vestibular nuclei^21–23^ and this multisensory neural processing is also found in multiple self-motion sensitive cortical areas^24,25^. Thus, multisensory information is tightly integrated in the nervous system and normally provides complementary or redundant information about our self-motion. The result of this process is sufficiently accurate to enable humans and animals to function day to day. The underlying process of constructing an estimate of traveled distance requires transducing basic sensory information and integrating this into a single estimate. When this sensory information is put into experimental conflict, somatosensory information can influence percepts arising from vestibular sensation for both static orientation^26^ and self-motion^27,28^. Humans and animals have evolved to perform these tasks within a constant one-g gravitational field, and thus the process of integrating haptic cues, and vestibular information in particular, may be disrupted as one moves away from the normal Earth environment. A number of studies have explored the impact of both short- (up to 22 s in parabolic flight) and long- (a few days to a several months) duration microgravity conditions on the perception of self-motion as it is of particular interest in terms of the overall operational performance of astronauts due to the differences in cues normally available during training on Earth^6,8,29–31^.

Although self-motion perception normally results from a multi-modal cue integration process where physical motion cues are integrated with visual cues^32^, visual movement alone (optic flow) can induce a compelling sense of self-motion known as vection^33^. Calculating the distance traveled from optic flow is part of the process of path integration, in which the course of an extended movement is estimated by integrating short pieces of the movement to yield the total path^34,35^.

From the earliest days of human space flight there have been concerns about the possible effects of microgravity on self-motion^29^. Studies have shown systematic changes in the perception of vection as a consequence of short-duration microgravity using microgravity aircraft^6^ and short duration (one to two week) space missions^7^. A wide range of perceptual errors have been reported when moving in microgravity. For example, onset latencies for the perception of self-motion are decreased^31,36^. One fundamental problem for the human perceptual system when estimating translation in unusual gravity situations is the disambiguation of ambiguous graviceptor (primarily the otoliths) cues. Graviceptor signals indicate both tilt with respect to gravity and acceleration of translational motion. Under normal gravity conditions the nervous system must disambiguate between head/body tilt relative to gravity and linear acceleration of the head/body. The process of this disambiguation relies on signals from a wide range of different perceptual cues and inherent assumptions^37^. Visual and haptic cues, along with the semicircular canals^38,39^ can provide information that may help in this disambiguation under Earth-normal conditions. Exposure to a new gravity state leads to a deterioration in the nervous system’s ability to disambiguate tilt from translation resulting in errors in both the perception of tilt and of translation^40–42^.

Unfortunately, little is known about the effects of long-duration microgravity on the perception of self-motion. To date, self-motion studies in microgravity have typically dealt with participants who have been subjected to one to two weeks of microgravity and have had to contend with small participant pools and limited visual stimuli. For example, Young et al.^7,43,44^. concluded that astronauts are more visually dependent in microgravity as they generally experience a stronger sense of rotational vection around the naso-occipital axis than they do on Earth. In one condition that is particularly relevant to the present study, Young et al.^7,43–45^. modulated the tactile cues to self-motion by loading the muscles of the participant’s lower limbs with bungee cords between their waist and the spacecraft’s floor. This tactile and proprioceptive loading simulated the weight normally carried by the lower limbs in normal gravity and significantly reduced participants’ reported circular vection. Similar results of the Neurolab mission demonstrated that latency of vection decreased and its perceived magnitude increased in microgravity^31^. More quantitative results for linear vection in long-duration microgravity exposure are not yet available, although this question is being probed by an ongoing experiment with the Canadian Space Agency (CSA ‘Vection’) on the ISS^46^. Similarly, little is known of the effect of buoyancy on the perception of vection. A recent study reports differences between vection on land and during buoyancy^47^ and raises the question as to whether neutral buoyancy might introduce unintended consequences in terms of the perception of self-motion relative to Earth-normal conditions.

**Fig. 1:**
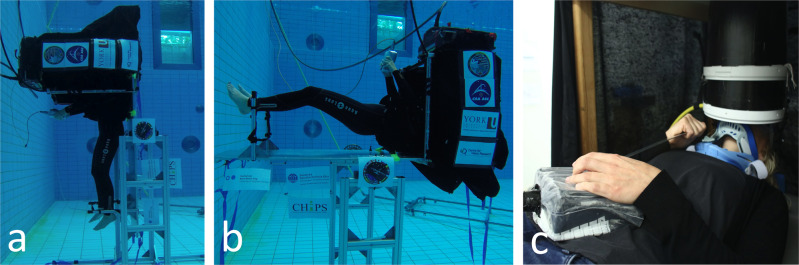
Experimental data collection. **a** Shows upright data collection in the pool, **b** Shows supine data collection in the pool, **c** Shows in-lab supine data collection with a closeup of the cylindrical viewing tube. Participants consented to the publication of the photographs.

In order to assess the potential impact on vection of the lack of normally sensed somatosensory cues created by neutral buoyancy and any possible interactions with body posture since it has been found to impact the perception of linear vection^48,49^, we conducted experiments with participants who were both neutrally buoyant in a pool and under Earth normal conditions in the lab in both supine and upright body postures with regard to the gravitational vertical (see Fig. 1).

We used three tests to assess perception of linear visual self-motion in a head-fixed visual display: (1) Move-to-Target^50^ in which participants were first presented a visual display that simulated moving forward at a constant acceleration towards the location of a previously presented target at different distances and indicated when they felt that they had moved through that distance; (2) Adjust-Target^51^ in which participants were first presented simulated forward self-motion through a given distance and subsequently adjusted the position of a target in the same environment to match the previously experienced distance; and (3) self-reported binary “vection experience” score (“yes” if the participant perceived vection or “no” if the participant did not). An additional size constancy control experiment was performed to determine if any difference in results by condition could be explained by differences in spatial compression of space between conditions as a consequence of the use of virtual reality.

We choose to use two different measures of vection magnitude (move-to-target and adjust-target) given the differences encountered between these two measures. See Lappe et al.^51^ for a description of the two measures and an explanation for their differences in terms of a leaky integrator model.

## Results

### Self-motion perception experiment

**Fig. 2:**
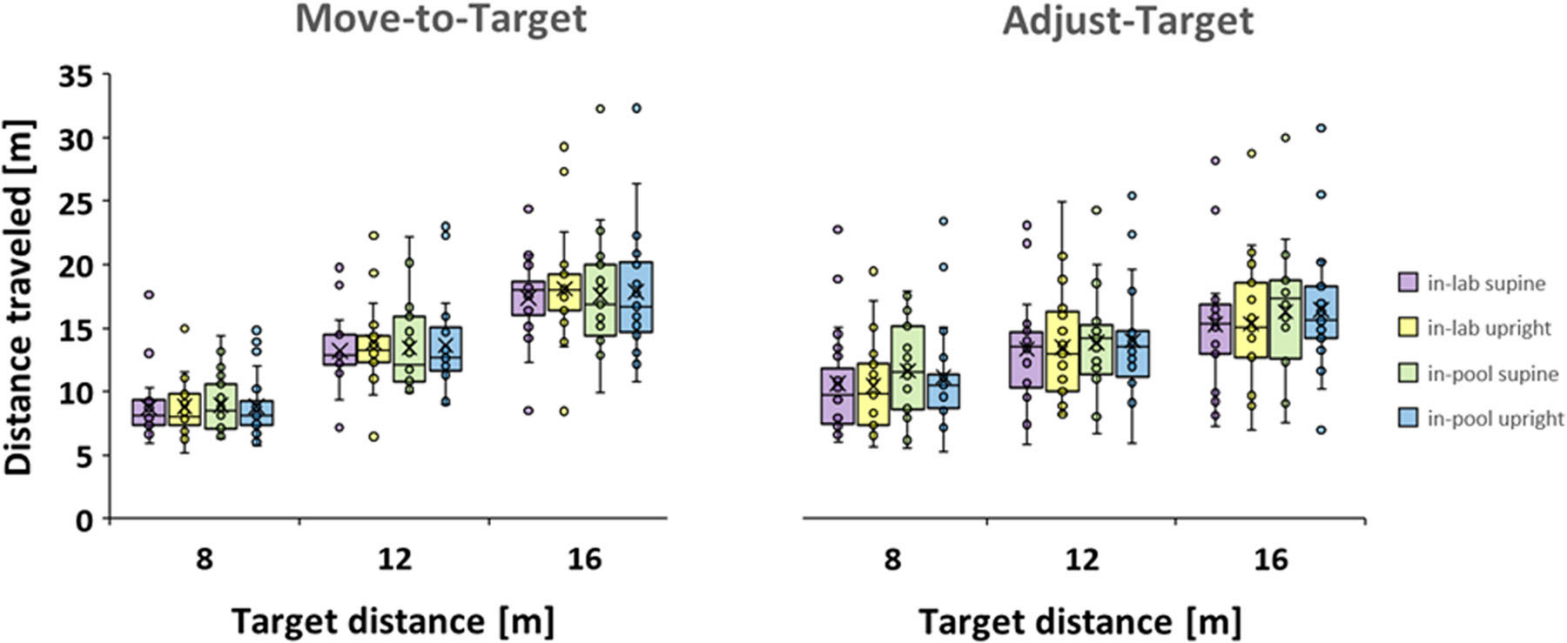
Travel distances. Distances needed for participants to perceive they had moved to the position of each of three previously seen target distances (Move-to-Target task, left panel) and the distance set as matching their previously experienced travel to those target distances (Adjust-Target task, right panel) for in-lab (purple and yellow bars) and in-pool (green and blue bars) for supine (purple and green bars) and upright (yellow and blue bars) postures. Targets were simulated at 8, 12 and 16 m from the participant (see Fig. 8a). Boxes represent ±1 interquartile range, whiskers ±1.5 interquartile range, lines are medians and “x” are means. A repeated measures ANOVA yielded significant effects for the factors task, task × distance and distance × posture.

Figure 2 plots mean travel distances reported from the Move-to-Target and Adjust-Target tasks (task) for the in-pool and in-lab conditions (buoyancy) for supine and upright postures (posture), for the three target distances (distance).

A repeated measures ANOVA was performed on task (2) × distance (3) × buoyancy (2) × posture (2). Significant differences were found for distance F(2,44) = 353.612, *p* < 0.001, ${\eta }_{p}^{2}$ = 0.941, for task × distance F(1.255, 27.605) = 35.283, *p* < 0.001, ${\eta }_{p}^{2}$ = 0.616, and for distance × posture F(2,44) = 3.314, *p* = 0.046, ${\eta }_{p}^{2}$ = 0.131. None of the other main effects or interactions were found to be significant. That is, there was no effect of whether the participants were underwater or not. The complete results of this and subsequent analyses are provided in the Supplementary Table S2 and Supplementary Fig. S1.

**Fig. 3:**
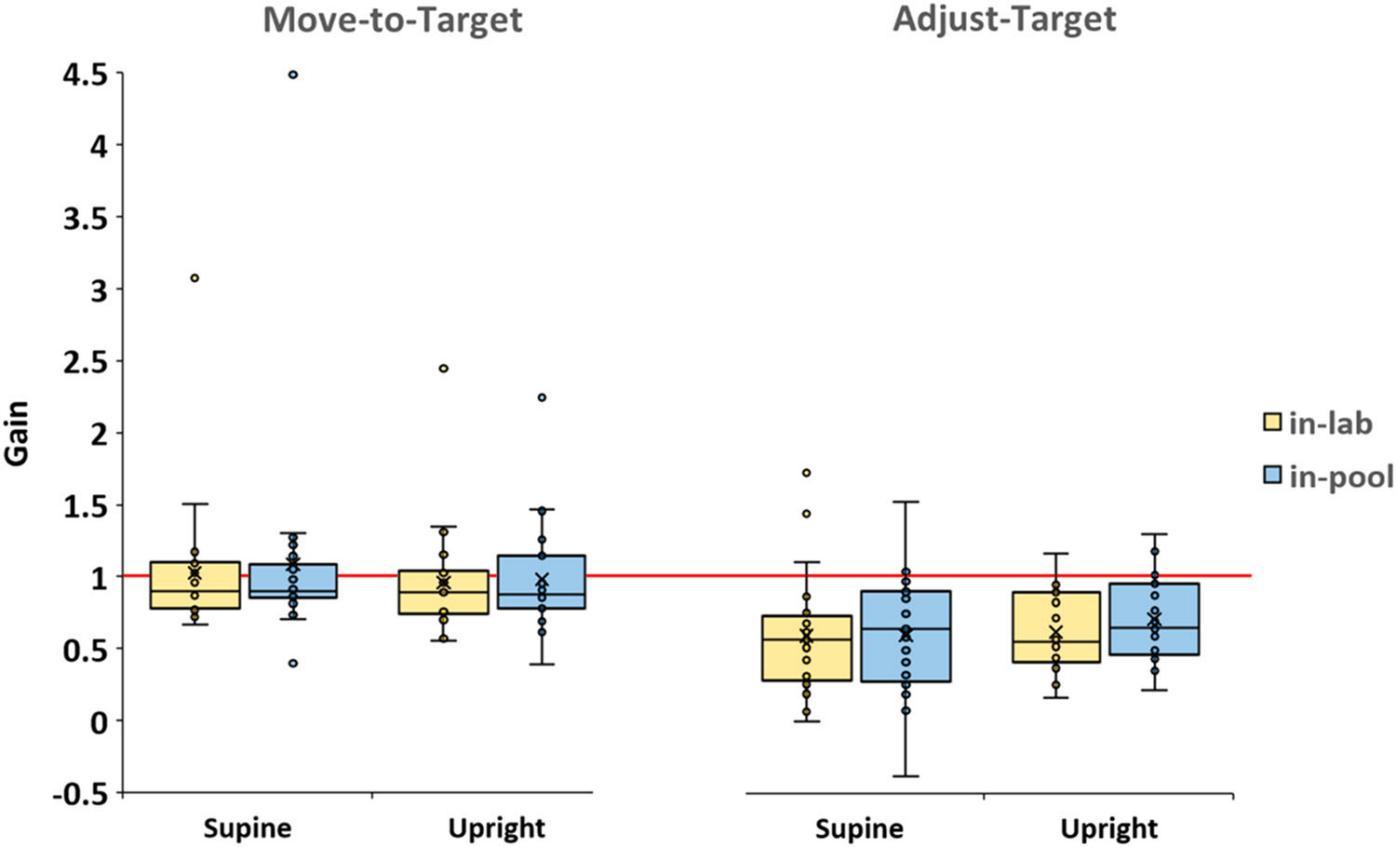
Perceptual gain. Gains for the Move-to-Target (left panel) and Adjust-Target (right panel) tasks are organized by supine versus upright x in-lab (yellow bars) versus in-pool (blue bars). Horizontal red line shows unity gain (perfect performance). Boxes represent ±1 interquartile range, whiskers ±1.5 interquartile range, lines are medians and “x” are means. A repeated measures ANOVA of task (2) × buoyancy (2) × posture (2) yielded significance only for the factor task.

A repeated measures ANOVA of the participants’ perceptual gain (target distance/participant response distance) was performed on task (2) × buoyancy (2) × posture (2) (see Fig. 3). A significant effect was found of task F(1,22) = 10.230, *p* < 0.004, ${\eta }_{p}^{2}$ = 0.317. There were no other significant effects (see Supplementary Table S3).

**Fig. 4:**
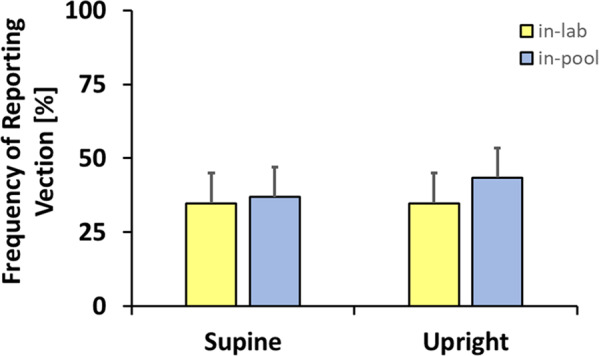
Vection experience. The percentage of time participants self-reported experiencing vection in-lab (yellow) and in-pool (blue) by posture. Error bars show standard errors (s.e.m.). A repeated measures ANOVA showed no significant effects.

Many studies exploring vection utilize self-reporting as their measure of the effectiveness of the visual stimulus in evoking the perception of self-motion. Figure 4 plots the percentage of times participants self-reported experiencing vection by body posture and buoyancy averaged over the Move-to-Target and Adjust-Target tasks and over target distance. A repeated measures ANOVA of participants’ self-reported vection was performed on buoyancy (2) x posture (2). There were no significant effects (see Supplementary Table S4 and Supplementary Fig. S2).

### Size constancy experiment

**Fig. 5:**
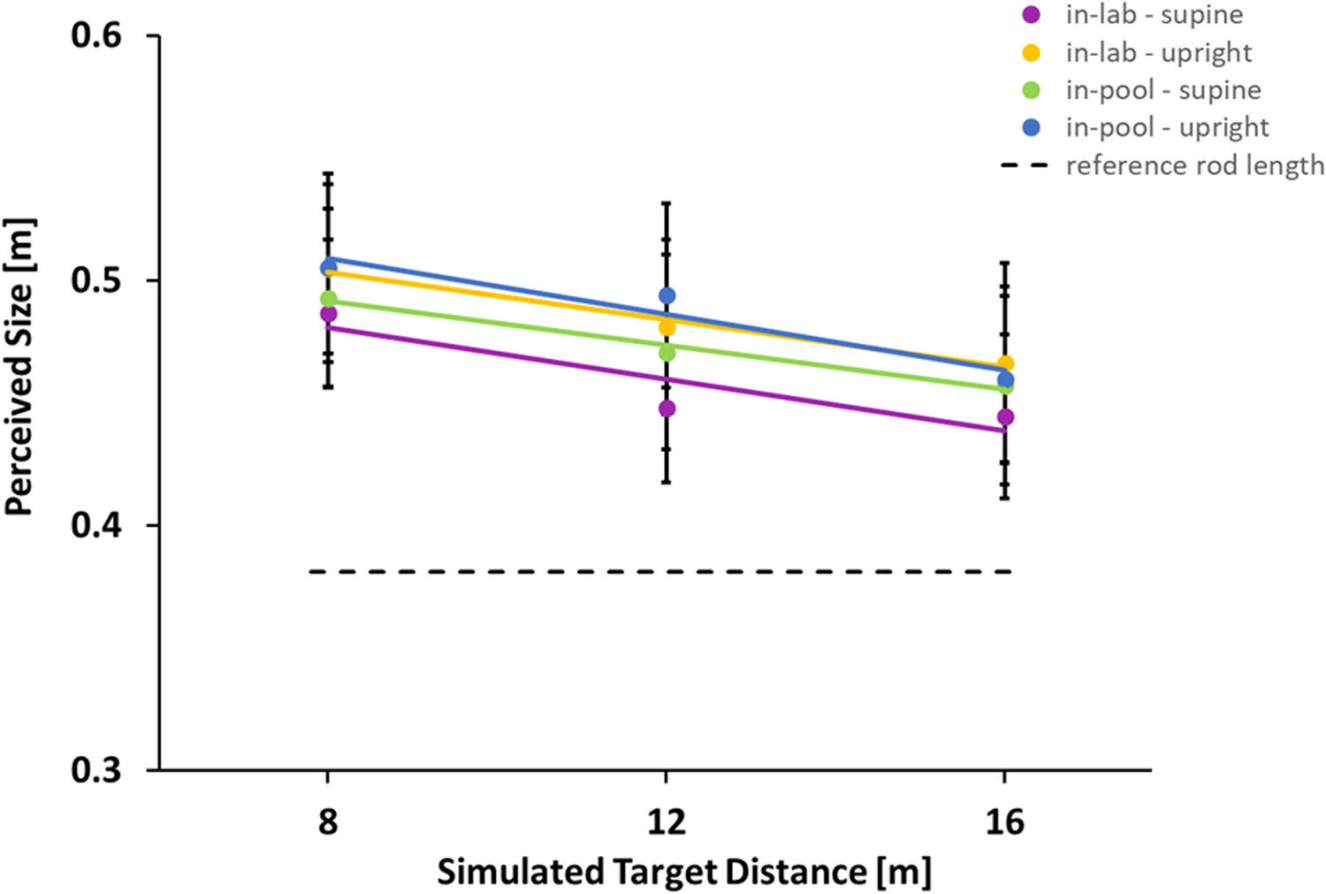
Size perception. The size of the target set as equal to the reference stick for supine (purple and green) and upright (yellow and blue) postures for in-lab (purple and yellow) and in-pool (green and blue) conditions. Error bars are standard errors (s.e.m.), lines are linear regression fits, and the actual length of the reference stick is indicated by the horizontal dashed line.

Figure 5 plots average size of the displayed stimulus that was set as matching the reference length as a function of the simulated distance of the targets for the four combinations of body posture and buoyancy. A repeated measures ANOVA of the participants’ size settings was performed across distance (3) × buoyancy (2) × posture (2). There were no significant effects. Supplementary Table S5 in the Supplementary Material summarizes the analysis.

## Discussion

Estimating distance traveled during self-motion is an inherently multisensory task. Previous or concurrent exposure to visual self-motion can influence haptically sensed distance traveled^52^ or the ability to maintain position while walking in place;^53^ conversely active and passive somatosensory and proprioceptive stimulation can modulate the strength of visual self-motion^54,55^. The current experiments assessed the impact of reduced somatosensation due to immersion on judgments of distance traveled based on visual self-motion. Although earlier experiments^50,51^ have demonstrated the ability to move to the location of a previously viewed target and to adjust a target to indicate a previously traveled distance to assess a participant’s perception of forward linear vection, this experiment found no statistically significant difference in participant performance between earth normal conditions and under reduced sensory cues while SCUBA diving submerged. SCUBA divers are known to be subject to disorientation effects, especially when inexperienced^56^. Our stimuli were effective in evoking the sensation of vection in around 40% of our participants (Fig. 4) but we found no significant difference in vection between in-lab (not neutrally buoyant) and in-pool (neutrally buoyant) conditions as measured through either self-reported vection or the results of the Move-to-Target or Adjust-Target tasks.

Why were no differences in vection found underwater? Self-motion is a multisensory event in which vestibular, visual, proprioceptive, somatosensory and motor signals are combined to allow successful navigation^32^. Microgravity removes somatosensory and vestibular cues whereas neutral buoyancy removes only somatosensory cues. Our study found no statistically significant difference in vection in removing somatosensory cues to posture. In contrast, a recent study^47^ reports that participants had a stronger sense of vection and felt that they had moved farther when buoyant and prone, than when standing upright out of the water. As posture has been found to impact the perception of linear vection^48,49^ and because of the nature of the stimuli they used, it is difficult to attribute these results to buoyancy. In contrast, we conclude that removing somatosensory cues to orientation has no statistically significant effect on the perception of self-motion.

Fauville et al.^47^ compared vection when neutrally buoyant and prone versus standing upright while observers watched a 5 min long virtual-reality movie which depicted over 270 m of non-straight-ahead motion at a non-constant velocity. This study found a significant enhancement of vection in the buoyant condition relative to the ground-based conditions. The study here did not find this significant effect, but there are significant differences between the studies; the study here using controlled linear motion at constant acceleration, over much shorter distances, with a controlled-visual display, and with upright and supine participants. However, in neither experiment described here were tactile cues from the surrounding completely eliminated, e.g. participants stood on the ground obtaining tactile cues from the feet and underwater, all participants were lightly tethered which may have provided somatosensory cues that have been known to influence vection^45^, and in our experiment the participants were also lightly tethered to an Earth-fixed display.

What happened to the compression of space? Our participants viewed the stimuli in the in-pool condition through a small amount of water (~35 cm) and the display would have been slightly distorted by the refraction of light from the display passing through the participants’ SCUBA goggles, the water column and the transparent viewport of the display^56,57^. Figure 5 and the statistical results shown in Supplementary Table S5 show no significant difference between the in-pool and in-lab data collection sessions in judging the size of a target – no significant magnification or perceptual effects from being submerged were observed.

**Fig. 6:**
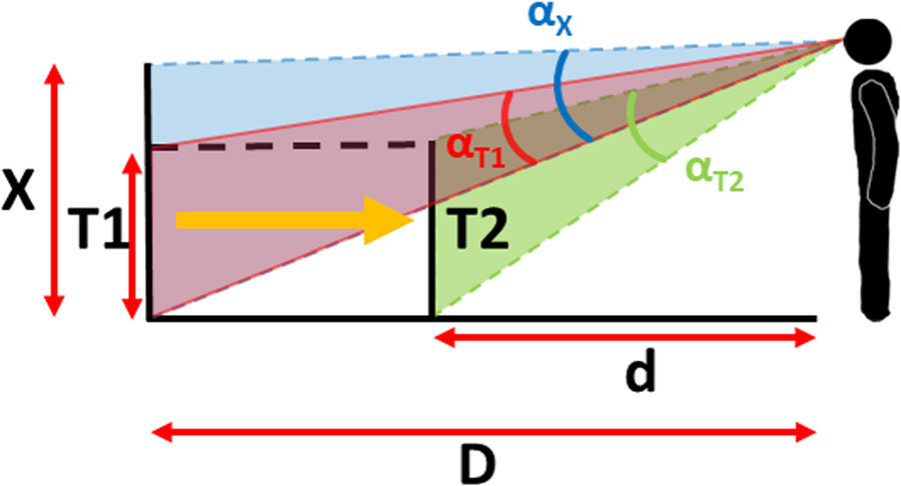
Size/distance equivalence. Illustration of the relationship between perceived size and perceived distance. The participant is seeing a target (T) at a simulated distance (D) which perceived size had to be adjusted to the length of the reference stick holding in his hands (not shown). If the target’s position were misperceived as closer (at distance d, shift shown by the large orange arrow), then it would be expected to subtend a larger visual angle (α_S_ > α_T_) and hence project a larger retinal image size (shown by the green triangle). To match this larger retinal image size, the target would need to be made larger (set to size X) so that its retinal image size (shown by the blue triangle) matched the expected retinal image size (green triangle) of the closer target, i.e. α_X_ = α_S_.

The design of this experiment used perceived size to reveal possible differences in perceived distance exploiting the phenomenon of size-distance equivalence^58,59^. In other words, if an object at a given distance needs to be set as larger than it really is to match an external reference length, then this may be because it is perceived as closer (Fig. 6). Figure 5 shows that all judgments were larger than accurate; a judgment compatible with all targets being seen as closer than they were – independent of the condition tested. Figure 6 shows how setting target size (X) as larger than the reference stick (S) is compatible with the target being seen as closer (d) than its simulated distance (D), under the assumption that size perception is constant.

This overall tendency to underestimate distances may be a consequence of using virtual reality which is known to lead to distance compression^60–63^, although the reasons for this are not yet fully understood. However, whether we consider these data in terms of distance or size the main observation relevant here is that there was no effect of submersion. Depth perception may be altered in short-duration microgravity^37^ and long-duration spaceflight^64^ although these measures have been rather indirect. Therefore, we are reporting another potential difference between the effect of neutral buoyancy and space on human perception: not only did we find no statistically significant effect of submersion on gains or perceived distance evoked by optic flow, nor did we find a statistically significant effect on perceived size/distance.

In this study, we identified four potential limiting factors - 1) the participant pool size, 2) the magnification effect of SCUBA goggles underwater, and 3) unintended somatosensory cues, and 4. self-reported vection.

1. Participant pool size. 23 participants took part in this study. One concern with the participants is that they were all experienced SCUBA divers. This was a requirement to minimize the risk to the participants as half of the data was collected while the participant was underwater. The use of experienced SCUBA divers may make these participants more adapted to the underwater environment and thus reduce the possible impact of buoyancy on vection. All of the participants completed the study and none reported issues that might have required their data collection session to be interrupted. Given the participant pool size in Fauville and colleagues’ study^47^ (20 in the buoyant condition and a different 18 in ground condition) and the large size of the effect found in Fauville and colleagues’ study, we would have expected to have seen a similar effect with 23 participants in a repeated measures study design. Nonetheless, we recognize that we might be missing effects due to the low number of data points (type II error) and look forward to replicating and extending this work in the future.

2. Magnification effect of SCUBA goggles underwater. SCUBA goggles introduce a magnificent effect due to the refraction of light through the goggles. The total refraction depends on two distances (object-to-faceplate and eyes-to-faceplate) which could have been taken into account. Previous studies (Luria & Kinney 1967; Ross, 1967; Franklin et al., 1970; Kinney & Luria, 1970; Ross et al. 1970; Goeters 1975; Ross & Nawaz, 2003) found that magnification index varied between 1.0 to 1.25 (depending on diver’s experience, adaptation to the condition and differences in experimental designs). We did not measure this magnification effect directly, but rather judged any impact on the perception of 3d size.

3. Unintended somatosensory cues. Although the goal of the buoyant condition was to remove all somatosensory cues, this was not completely achievable in practice. Motion in all directions was constrained by the participants wearing a BCD (Buoyancy Control Device) diving jacket which was filled with air to maintain neutral or slightly positive buoyancy and to prevent them from touching the metal scaffold. The BDC jacket was loosely strapped to the metal scaffold. In the supine position the participant floated about 15 cm above the metal scaffold. In the upright position the participants also floated about 15 cm sideways from the metal scaffold (see Fig. 6a, b). The participant also floated with their head touching the cylindrical viewing tube. These and other unintended somatosensory cues may have contributed to a modulation of linear vection, as reported for circular vection by Young and colleagues^7,43–45^.

4. Self-reported vection. Participants completed a short questionnaire that sought to capture the level of immersion as measured by the participant’s perceived vection. One constraint here that complicated direct estimates of vection was the difficulty of introducing detailed questionnaires after each experimental trial as the participant was loosely secured in the apparatus at depth for all of the neutral buoyancy conditions. It was not practical to obtain self-reported vection scores after each trial or indeed after each session. Given that reporting was only possible after the entire buoyant session, the same self-reported binary “vection experience” score was collected under Earth-normal conditions for consistency.

In conclusion, preparing astronauts for the rigours of outer space and the debilitating effects of microgravity requires analog environments to train for the novel environment and to rehearse tasks that will be later performed on orbit. A range of Earth-based analog environments have been developed including short-duration microgravity flight, head down bed rest, isolation environments, human centrifugation and neutral buoyancy tanks. Here we were unable to demonstrate that neutral buoyancy impacts the perception of self-motion relative to ground-based performance. Furthermore, we were unable to demonstrated a change in the compression of space found in ground-based virtual environments when a participant is buoyant. This suggests that vection-related tasks will not be impacted relative to their Earth-based performance when performed in a neutral buoyancy tank if similar visual cues are provided. If future data reveal differences between self-motion perception as a consequence of long duration space missions and on Earth, our present study has shown that these would most likely be ascribable to vestibular dysfunction – the lack of somatosensory cues alone does not compromise the perception of self-motion.

## Methods

### Participants

Twenty-three right-handed participants (10 female, 13 male; 25.5 ± 6.1 yrs.) took part in this study, which was pre-approved by the ethic committees of York University and the German Sport University Cologne and conformed to the Declaration of Helsinki (1964). All participants were experienced SCUBA divers; They reported more than 20 h of practical SCUBA training as well as more than 20 h of theoretical training about diving and between 5 and 150 dives outside of a pool. Participants reported normal or corrected-to-normal vision and reported no vestibular, tactile or somatosensory dysfunction by self-report. Participants reported no prior experience in research on self-motion perception. Experienced divers were selected as they had the necessary experience to participate safely in this study. Each participant read and signed an informed consent statement before testing began. Participants were recruited from the German Sport University of Cologne and from local dive clubs in the Cologne region of Germany. Participants received no compensation for their participation in this study.

### Conditions

Participants were tested in two environmental conditions (in a laboratory and underwater in the German Sport University Swimming Facility in Cologne, Germany) and in two body postures (upright and supine). The order in which a participant experienced the environmental condition was randomized but once in that environment the participant completed all of the in-pool or all of the in-lab data collection sessions in a group. The order of the body postures was randomized within either the in-lab or the in-pool group.

### Equipment

Outside the water, stimuli were displayed on an LCD panel (HP Compaq 1520) and inside the water, the same stimuli were displayed on a different but similar LCD panel mounted in a custom water-proof case. In both cases the display was connected to a HP Zbook Studio G3 equipped with a NVIDIA Quadro M1000M video card running Windows 7 Professional. The display had a resolution of 1024 × 768 and was viewed through a cylindrical tube (diameter 22.5 cm, length 32 cm) made of black/gray plastic (Fig. 1). Viewing distance was 35 cm controlled by the length of the viewing tube. The display was viewed binocularly. Participants responded using a custom USB controller that was designed to be waterproof. The same computer and controller were used for both the in-pool and in-lab data collection sessions.

For both the in-pool and in-lab data collections external visual stimuli were blocked with a shroud. For in-pool sessions a metal scaffold was used to loosely restrain the participant in the apparatus, while for in-lab sessions a mechanical structure was used to support the viewing cylinder and display. For in-lab sessions participants wore a cervical collar while during in-pool sessions participants wore a strap that fixed their head motions relative to the body. For in-pool sessions participants wore full 7 mm wetsuit SCUBA equipment excluding fins. During in-lab sessions participants breathed through their regulator and, to ensure a common field of view between in-pool and in-lab conditions, wore SCUBA goggles with the lenses removed during in-lab data collection.

### Virtual reality

**Fig. 7:**
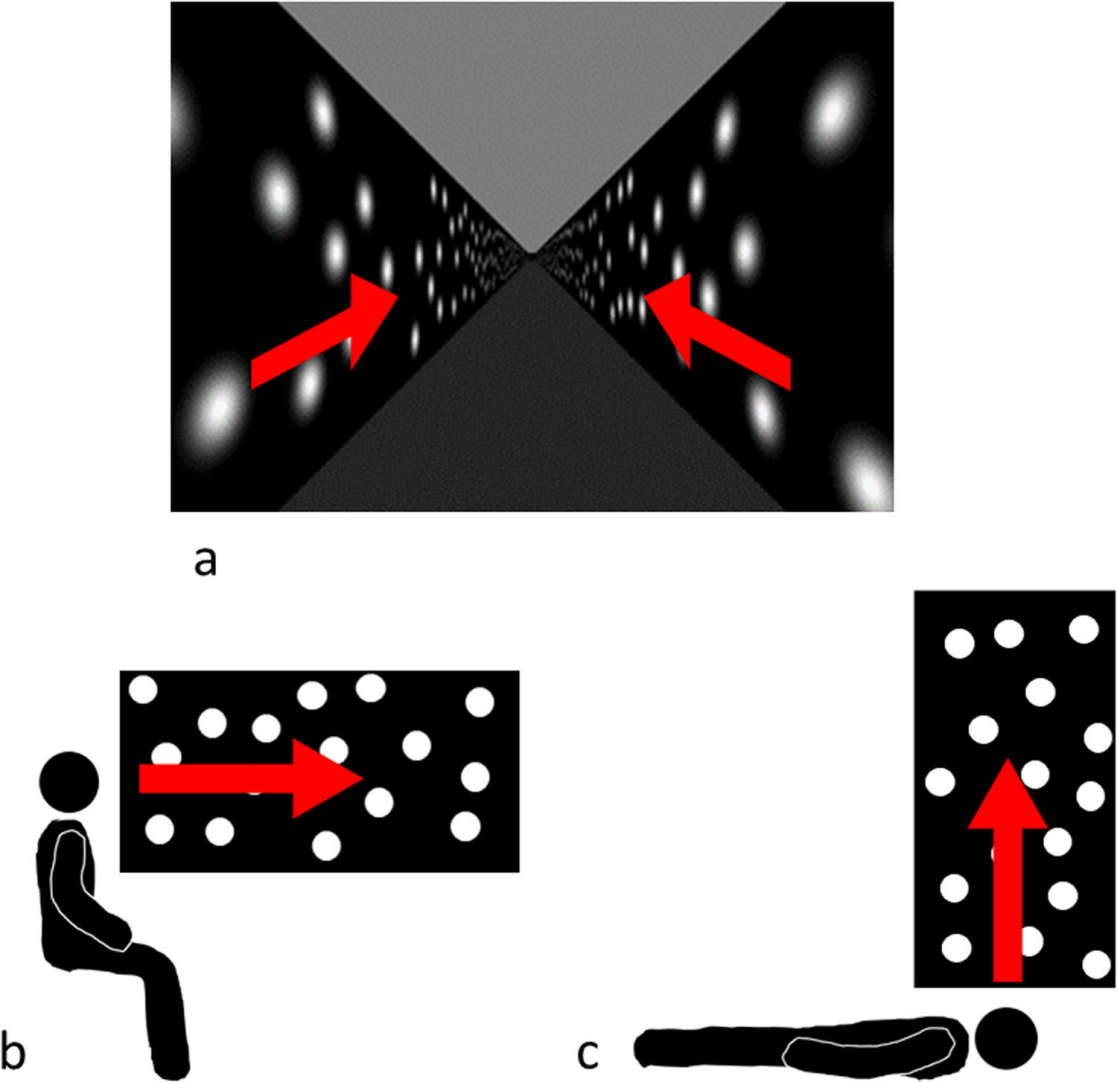
Direction of simulated motion. Red arrows show direction of simulated motion in (**a**) the virtual reality (i.e. hallway), (**b**) the sitting upright condition, (**c**) the lying supine condition. Simulated motion is perceived along the participants’ sagittal axis in both sitting upright and lying supine (i.e. participants’ vision was head-fixed).

**Fig. 8:**
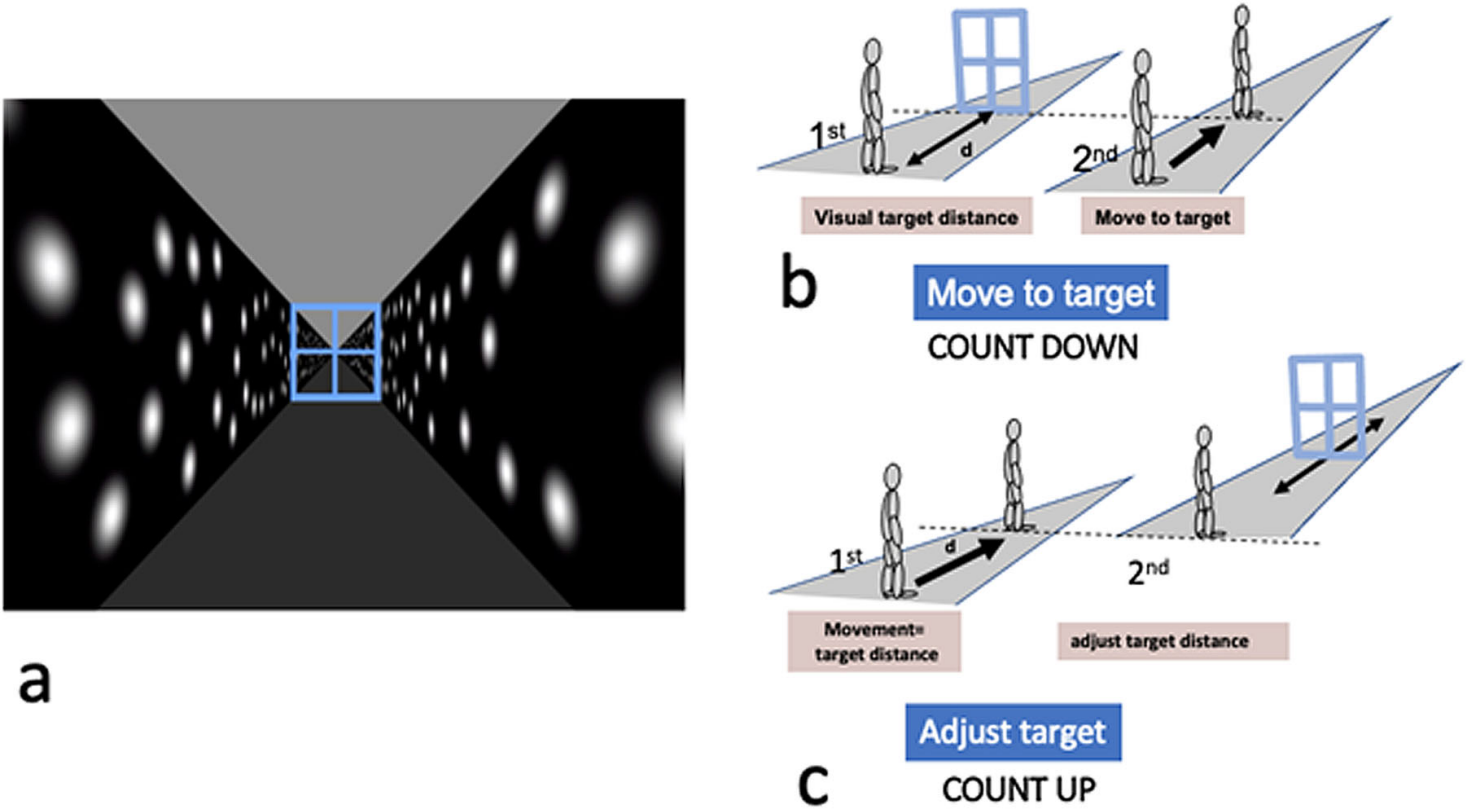
The Move-to-Target and Adjust-Target tasks. **a** Shows the view down the simulated hallway. The Gaussian blobs on the walls flickered at a random rate. The target filled the corridor. **b** Illustrates the participant’s task in the Move-to-Target task. A target appeared at some distance down the corridor. The target was then extinguished, and the participant is moved visually down the hallway. Participants pressed a button on the keypad to indicate when they have reached the position of the previously presented target. **c** The participant’s task in the Adjust-Target task. The participant was first moved visually down the hallway through a predetermined distance. The target then appeared in front of them, and the participant adjusted its position using the keypad so that it appeared at the distance through which they perceived themselves to have just moved. The participant then pressed a button on the keypad to confirm this distance and the next trial began.

Participants viewed a simulated environment with their head fixed 1.65 m above the floor centered within a simulated 3.3 m wide square hallway aligned with the body that stretched out in front of them to infinity along the sagittal axis. Participants perceived the simulated motion through this virtual hallway along this axis in both sitting upright and lying supine (i.e. participants’ view was head-fixed) (see Fig. 7). No fixation cross was present at any time of the experiment (i.e. there was no fixation marker for any of the three tasks), but participants were asked to look down the hallway during the Move-to-Target and Adjust-Target tasks. The ceiling and the floor of the hallway were colored gray and the walls colored black (see Fig. 8a). Random white Gaussian texture blobs (diameter = 0.8 m, sigma = 0.2 m) were presented on the walls of the hallway. These blobs appeared and disappeared on a random schedule to reduce the possibility that a participant might track one of these cues rather than process the entire visual field. There was no stereo generated in the display and participants were not able to move their heads to obtain parallax cues.

For each combination of environmental condition and body orientation participants performed three tasks that were presented in the sequence: *Move-to-Target, Adjust-Target*, and *Size Constancy*.

For the first task (Move-to-Target), the time of exposure to visual flow was dependent on the participants individual perception of motion, i.e. the more motion they felt, the earlier they pressed the button which then stopped the motion. For the latter task (Adjust-Target), the time of exposure of visual motion was automatically stopped after 4.472 s (8 m), 5.477 s (12 m), 6.325 s (16 m), which is based on simulated motion being presented with an acceleration of 0.8 m/s^2^. The time participants needed to finish the three tasks were about 3:30 min for the Move-to-Target task, 6:30 min for the Adjust-Target task, and 3 min for the Size Constancy task. Overall, participants were exposed to each environmental condition for about 19–23 min (3–5 min for preparation, 13 min for doing the tasks, 2 min for changing their posture, 1–3 min for getting out of the equipment).

### Move-to-Target task

In each trial the participant viewed a visual simulation of a corridor. Within the visual simulation, they viewed a target that filled the corridor presented at one of three simulated distances (8 m, 12 m, 16 m). The participant was asked to view the target and build an internal estimate of its distance from the perspective cues. A typical view is shown in Fig. 8a. When ready, the participant pressed a button on the keypad and the target was extinguished and the participant was subjected to simulated visual motion towards the previously presented target position at a constant acceleration of 0.8 m/s^2^. Motion was stopped as soon as participants indicated when they thought they had reached the position of the previously presented target by pressing a button. The structure of the task is summarized in Fig. 8b.

### Adjust-Target task

This task was similar to the Move-to-Target task with one crucial difference. In the first part, the participant’s viewpoint was moved through one of three distances (8 m, 12 m, 16 m - the same as the target distances as in the Move-to-Target task) and at the same acceleration as in the Move-to-Target Task (0.8 m/s^2^). Following the movement, a target appeared (the same target as used in the Move-to-Target Task, Fig. 8a) and the participant adjusted the target’s position to indicate the distance through which they had just traveled by means of keypad buttons. The structure of the task is summarized in Fig. 8c.

### Size Constancy task

**Fig. 9:**
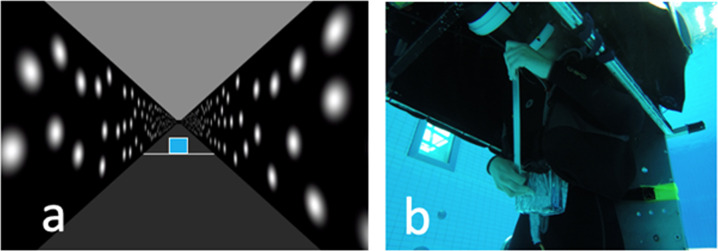
Size Constancy task. **a** Shows the participant’s view in the display, **b** Shows the participant holding the stick in the upright body posture in the in-pool condition. Participant consented to the publication of the photograph.

In this task participants judged the height of a front-facing square polygon relative to a reference stick that they held in their hands aligned with the long axis of their body. Participants viewed presentations of the square in the same visual hallway as used in the previous two tasks (Fig. 9a) at 8 m, 12 m and 16 m. For each presentation of the target square, participants judged if it was taller than, or shorter than a physical stick that they held aligned with their long body axis (Fig. 9b). The stick was 38.1 cm (15”) long. In this task, no simulated motion was presented.

### Reporting summary

Further information on research design is available in the Nature Research Reporting Summary linked to this article.

## Data analysis

All valid data (using the criteria described below for each task) were used for the statistical analysis (repeated measures ANOVAs). For tests within which Mauchly’s Test of Sphericity was violated, Greenhouse-Geisser correction was performed on the degrees of freedom for the analysis. Post-hoc t-tests with Bonferroni correction were performed on each of the significant effects. All statistical tests were performed using SPSS 28 and the significance level was set to alpha = 0.05.

### Move-to-Target/Adjust-Target task

For each trial the visually simulated distance traveled was recorded – either the amount of optic flow required to reach the remembered position of the previously viewed target (Move-to-Target task), or the adjusted target’s position which matches the previously traveled distance (Adjust-Target task). Each distance was presented eight times in a pseudorandom order. Any observations that were more than 2σ away from the participant’s mean response for each distance for each body orientation/buoyancy condition were discarded. This data validation was performed separately for the Move-to-Target and Adjust-Target tasks. Supplementary Table S1 in the Supplementary Material summarizes the responses discarded and percentage of discarded responses. Subsequently, participants’ means of both tasks were used for further statistical analysis and the calculation of the gains (see below).

For the Move-to-Target task, we define the perceptual gain^50^ as the slope of the regression fit of perceived versus actual motion (target distance as a fraction of the distance traveled; output/input). The perceptual gain represents the amount of visual motion required for a given amount of perceived motion – the higher the gain, the less visual motion that is required to evoke the sensation of travel through a given distance. For the Adjust-Target task, the perceptual gain is the slope of the regression fit for the distance set versus the distance traveled (output/input)^51^.

#### Self-reported vection score

Following each data collection session (underwater and lab) participants reported their perceived vection (whether they had the sensation of moving) as either “no vection” or “vection” for both the Move-to-Target task and the Adjust-Target task as well as for both the supine posture and the upright posture.

### Size Constancy task

For each body posture/buoyancy/distance condition, the size of the stimulus was controlled by two interleaved psychometric staircases (PESTs)^65^. One staircase started with a square much larger (at 76 cm) than the stick length and one starting with the square much smaller (at 19 cm) than the stick length (see Supplementary Fig. S3). The six PESTs (two for each distance) were randomly interleaved. Each PEST was terminated after a maximum of 25 trials or when a total of 13 reversal responses were collected. Participant’s data was excluded from further analysis if the target’s height had hit either limit (0.095 m or 1.52 m) more than 5 times in a row. Based on this criteria, for the upright in-pool condition, a full dataset was not recorded for one of the participants for the Size Constancy task. This participant was therefore dropped from the Size Constancy task but retained for the other tasks (Move-to-Target and Adjust-Target) for which a full dataset was available.

Participants’ responses for a given target distance, in-pool/in-lab and body posture, were grouped and binned into one of 14 equal-width bins that spanned the minimum target size to maximum target size range. These bins enabled participant responses from −1 (target too small) and +1 (target too large) to be averaged over the bin. The resulting set of points were fit with a psychometric function as shown in Supplementary Fig. S3b. From this fit the participant’s perceived target size (the 50% point) was extracted.

## Supplementary information

Reporting Summary

Supplemental material

## Data Availability

The data that support the findings of this study are available in figshare with the identifier 10.6084/m9.figshare.15067548.
