# Supplementary material for: Vection underwater illustrates the limitations of neutral buoyancy as a microgravity analog
Source: NPJ Microgravity. 2023 Jun 10;9:42. doi: 10.1038/s41526-023-00282-3 (PMC10257652; doi:10.1038/s41526-023-00282-3)
Supplement: Supplementary file 1 — Reporting Summary [file 41526_2023_282_MOESM1_ESM.pdf]

nature portfolio

Corresponding author(s): DBPR

Last updated by author(s): May 9, 2023

Reporting Summary

Nature Portfolio wishes to improve the reproducibility of the work that we publish. This form provides structure for consistency and transparency in reporting. For further information on Nature Portfolio policies, see our Editorial Policies and the Editorial Policy Checklist.

Statistics

For all statistical analyses, confirm that the following items are present in the figure legend, table legend, main text, or Methods section.

n/a	Confirmed	
☐	☒	The exact sample size (*n*) for each experimental group/condition, given as a discrete number and unit of measurement
☐	☒	A statement on whether measurements were taken from distinct samples or whether the same sample was measured repeatedly
☐	☒	The statistical test(s) used AND whether they are one- or two-sided<br>*Only common tests should be described solely by name; describe more complex techniques in the Methods section.*
☒	☐	A description of all covariates tested
☐	☒	A description of any assumptions or corrections, such as tests of normality and adjustment for multiple comparisons
☐	☒	A full description of the statistical parameters including central tendency (e.g. means) or other basic estimates (e.g. regression coefficient) AND variation (e.g. standard deviation) or associated estimates of uncertainty (e.g. confidence intervals)
☐	☒	For null hypothesis testing, the test statistic (e.g. *F*, *t*, *r*) with confidence intervals, effect sizes, degrees of freedom and *P* value noted<br>*Give P values as exact values whenever suitable.*
☒	☐	For Bayesian analysis, information on the choice of priors and Markov chain Monte Carlo settings
☒	☐	For hierarchical and complex designs, identification of the appropriate level for tests and full reporting of outcomes
☐	☒	Estimates of effect sizes (e.g. Cohen's *d*, Pearson's *r*), indicating how they were calculated

Our web collection on statistics for biologists contains articles on many of the points above.

Software and code

Policy information about availability of computer code

Data collection	Unity v2017.4
Data analysis	SPSS 28

For manuscripts utilizing custom algorithms or software that are central to the research but not yet described in published literature, software must be made available to editors and reviewers. We strongly encourage code deposition in a community repository (e.g. GitHub). See the Nature Portfolio guidelines for submitting code & software for further information.

Data

Policy information about availability of data

All manuscripts must include a data availability statement. This statement should provide the following information, where applicable:

- Accession codes, unique identifiers, or web links for publicly available datasets
- A description of any restrictions on data availability
- For clinical datasets or third party data, please ensure that the statement adheres to our policy

The data that support the findings of this study are available in figshare with the identifier "https://doi.org/10.6084/m9.figshare.15067548".

Research involving human participants, their data, or biological material

Policy information about studies with human participants or human data. See also policy information about sex, gender (identity/presentation), and sexual orientation and race, ethnicity and racism.

Reporting on sex and gender	N/A
Reporting on race, ethnicity, or other socially relevant groupings	N/A
Population characteristics	See below (Research sample)
Recruitment	See below (Sampling strategy).
Ethics oversight	York University (Toronto, Canada), German Sport University Cologne (Cologne, Germany)

Note that full information on the approval of the study protocol must also be provided in the manuscript.

Field-specific reporting

Please select the one below that is the best fit for your research. If you are not sure, read the appropriate sections before making your selection.

☐ Life sciences ☒ Behavioural & social sciences ☐ Ecological, evolutionary & environmental sciences

For a reference copy of the document with all sections, see nature.com/documents/nr-reporting-summary-flat.pdf

Behavioural & social sciences study design

All studies must disclose on these points even when the disclosure is negative.

Study description	Experimental study with repeated measures in two postures and two conditions. Quantitative data.
Research sample	Twenty-three right-handed participants (10 female, 13 male; 25.5 ± 6.1 yrs.). Participants reported no vestibular, tactile or somatosensory dysfunction by self-report, and no such dysfunction were observed by the experimenters during testing They had no prior experience in research on self-motion perception. They reported more than 20 hours of practical SCUBA training as well as more than 20 hours of theoretical training about diving and between 5 and 150 dives outside of a pool.
Sampling strategy	Participants were recruited by word-of-mouth on campus of the German Sport University Cologne, particularly in the three SCUBA courses of that semester, and from local dive clubs in the Cologne region of Germany.<br>Sample size was selected to be within the range of our earlier pertinent studies
Data collection	Participants responded using a custom USB controller that was designed to be waterproof. Data was collected on a computer.
Timing	May 29th, 2018 to Feb 23th, 2023
Data exclusions	For the upright in-pool condition, a full dataset was not recorded for one of the participants for the size judgement task. This may have been the result of equipment failure and/or the participant being unable to complete the task. This participant was therefore dropped from the size judgement study but retained for other studies for which a full dataset was available.
Non-participation	No participant dropped out/declined participation.
Randomization	The order in which a participant experienced the environmental condition was randomized but once in that environment the participant completed all of the in-pool or all of the in-lab data collection sessions in a group. The order of the body postures was randomly assigned was also randomized within either the in-lab or the in-pool group.

Reporting for specific materials, systems and methods

We require information from authors about some types of materials, experimental systems and methods used in many studies. Here, indicate whether each material, system or method listed is relevant to your study. If you are not sure if a list item applies to your research, read the appropriate section before selecting a response.

Materials & experimental systems

n/a	Involved in the study
☒	☐ Antibodies
☒	☐ Eukaryotic cell lines
☒	☐ Palaeontology and archaeology
☒	☐ Animals and other organisms
☒	☐ Clinical data
☒	☐ Dual use research of concern
☒	☐ Plants

Methods

n/a	Involved in the study
☒	☐ ChIP-seq
☒	☐ Flow cytometry
☒	☐ MRI-based neuroimaging
