# Supplementary material for: Vection underwater illustrates the limitations of neutral buoyancy as a microgravity analog
Source: NPJ Microgravity. 2023 Jun 10;9:42. doi: 10.1038/s41526-023-00282-3 (PMC10257652; doi:10.1038/s41526-023-00282-3)
Supplement: Supplementary file 2 — Supplemental material [file 41526_2023_282_MOESM2_ESM.docx]

**Supplementary Material**

**Vection underwater illustrates the limitations of neutral buoyancy as a microgravity analog**

Nils-Alexander Bury^1,2,3^*, Michael Jenkin^2,4^, Robert S. Allison^2,4^, Rainer Herpers^1,4,5^ and Laurence R. Harris^2,3^

**Affiliations**

*^1^* Institute of Visual Computing, Hochschule Bonn-Rhein-Sieg, Grantham-Allee 20, 53757 St. Augustin, Germany

*^2^* Centre for Vision Research, York University, 4700 Keele St., Toronto, ON M3J 1P3, Canada

*^3^* Dept. of Psychology, York University, 4700 Keele St., Toronto, ON M3J 1P3, Canada

*^4^* Department of Electrical Engineering & Computer Science, York University, 4700 Keele St., Toronto, ON M3J 1P3, Canada

*^5^* Faculty of Computer Science, University of New Brunswick, Fredericton, Canada

***Corresponding author**

Nils-Alexander Bury

E-mail: [nils.bury@h-brs.de](mailto:nils.bury@h-brs.de)

Phone: +49 2241 865 712

**Outliers in Move-to-Target and Adjust-Target tasks**

Table S1 summarizes outliers in the Move-to-Target and Adjust-Target tasks. A response was considered an outlier if it was more than 2 sigma from the participant’s mean response for that condition (buoyancy, posture, target distance).

***Table S1: Outliers in the Move-to-Target and Adjust-Target tasks.***

|  | in-pool | | in-lab | |
| --- | --- | --- | --- | --- |
|  | Supine | Upright | Supine | Upright |
| Move-to-Target | 11 (2.0%) | 19 (3.4%) | 15 (2.8%) | 17 (3.1%) |
| Adjust-Target | 6 (1.1%) | 14 (2.5%) | 11 (2.0%) | 13 (2.4%) |

**Statistical tests**

Tables S2-S4 report the statistical results for repeated measures analysis of the Move-to-Target task and Adjust-Target task in the manuscript. Table S5 reports the statistical results for the repeated measures analysis of the Size/Distance Perception task related to perceived compression of space.

Post-hoc t-tests with Bonferroni correction were performed on each of the significant effects. For distances (plotted in Figure S1), the difference in response for all three distances were significantly different: for distances 8 and 12 t(183)=-26.057, p<0.003, for distances 8 and 16 t(183)=-27.612, p<0.003, and for distances 12 and 16 t(183)=-19.613, p<0.003. Given the significant main effect of distance, for method x distance and posture x distance, post-hoc tests were performed for the difference in methods and postures at the three distances separately. For the method x distance interaction, there was a significant difference in the interaction at 8m t(91)=4.065, p<0.003, but not at 12m t(91)=0.457 p>=1.00 n.s., nor at 16m t(91)=-2.393, p=0.057 n.s. For the posture x distance interaction, none of the distances were found to be significant; 8m t(91)=0.458, p>=1, 12m t(91)=0.441, n.s., nor at 16m t(91)=0.750, n.s. Table S2 and Figure S1 summarizes the results from the repeated measures analysis and plots of the interactions.

*
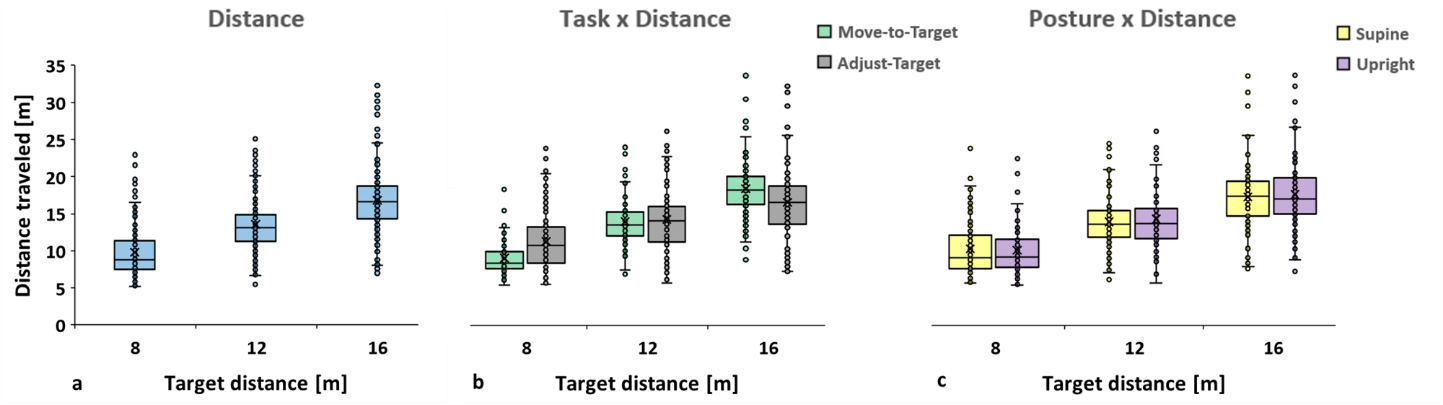
*

***Figure S1*** ***Post-hoc analysis of significant results from Table S2****. a) Distance judgements, b) Task x Distance (green using “Move-to-Target”; gray using “Adjust Target”), c) Distance x Posture (yellow “Supine”, purple “Upright”). Boxes represent ±1 interquartile range, whiskers ±1.5 interquartile range, lines are medians and “x” are means.*

***Table S2. Summary of repeated measures analysis of the Move-to-Target task and Adjust-Target task.***

|  | F value | Probability | $\eta_{p}^{2}$ |
| --- | --- | --- | --- |
| Task | F(1,22)=0.0354 | 0.853 | 0.002 |
| Distance | F(2,44)=353.612 | <0.001 | 0.941 |
| Buoyancy | F(1,22)=2.510 | 0.127 | 0.102 |
| Posture | F(1,22)=0.805 | 0.379 | 0.035 |
| Task x Distance | F(1.255,27.605)=35.283 | <0.001 | 0.616 |
| Task x Buoyancy | F(1,22)=2.088 | 0.163 | 0.087 |
| Task x Posture | F(1,22)=0.809 | 0.378 | 0.035 |
| Distance x Buoyancy | F(2,44)=0.382 | 0.685 | 0.017 |
| Distance x Posture | F(2,44)=3.314 | 0.046 | 0.131 |
| Buoyancy x Posture | F(1,22)=0.223 | 0.642 | 0.010 |
| Task x Distance x Buoyancy | F(2,44)=0.525 | 0.595 | 0.023 |
| Task x Distance x Posture | F(1.533,33.734)=0.047 | 0.954 | 0.002 |
| Task x Buoyancy x Posture | F(1,22)=0.080 | 0.780 | 0.004 |
| Distance x Buoyancy x Posture | F(2,44)=0.186 | 0.831 | 0.008 |
| Task x Distance x Buoyancy x Posture | F(2,44)=0.462 | 0.633 | 0.021 |

***Table S3. Summary of the analysis of visual gains.***

|  | F value | Probability | $\eta_{p}^{2}$ |
| --- | --- | --- | --- |
| Task | F(1,22)=10.230 | <0.004 | 0.317 |
| Buoyancy | F(1,22)=2.466 | 0.131 | 0.101 |
| Posture | F(1,22)=0.033 | 0.857 | 0.002 |
| Task x Buoyancy | F(1,22)=0.006 | 0.950 | 0.000 |
| Task x Posture | F(1,22)=2.780 | 0.110 | 0.112 |
| Buoyancy x Posture | F(1,22)=0.161 | 0.692 | 0.007 |
| Task x Buoyancy x Posture | F(1,22)=0.720 | 0.405 | 0.032 |

Correlating the gain for each task with the self-reported vection score which combines both the lying posture and the upright posture and both the underwater condition and the lab condition for each participant (Figure S2) indicated no significant effect.


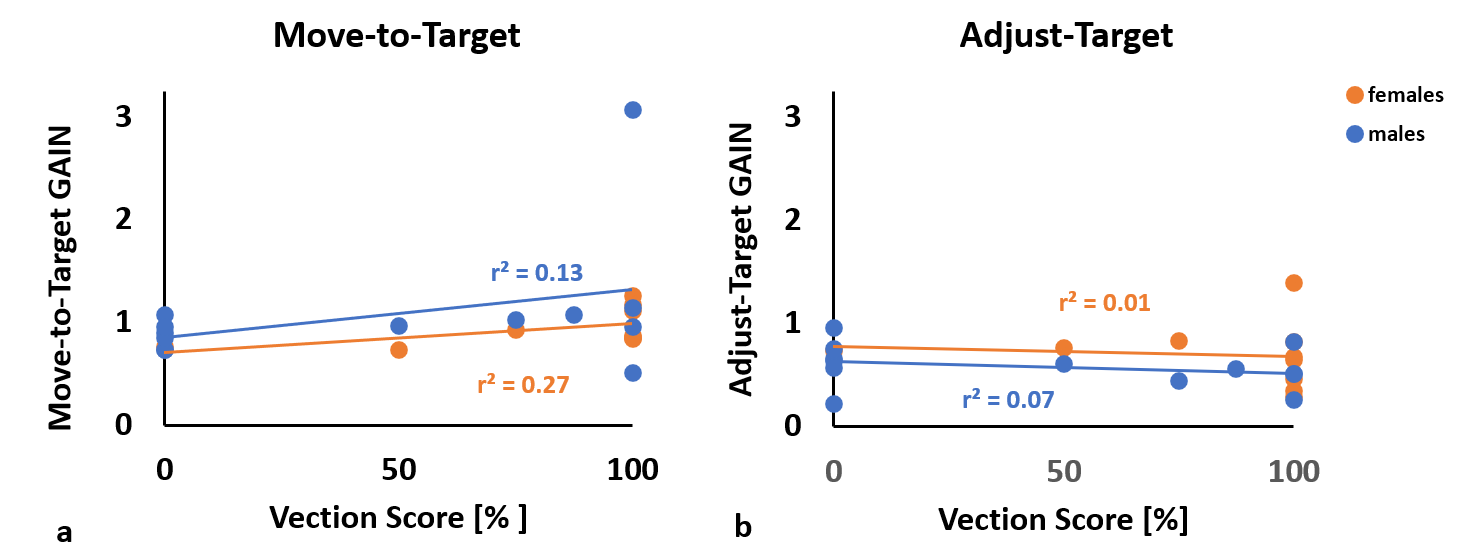


***Figure S2. Correlating self-reported vection****. Scores (each data point combines both the lying posture and the upright posture and both the underwater condition and the lab condition for each participant) with gain for the Move-to-Target (a) and Adjust-Target (b) tasks. Females: orange (r^2^=0.27 for Move-to-Target, r^2^=0.01 for Adjust-Target), males: blue (r^2^=0.13 for Move-to-Target, r^2^=0.07 for Adjust-Target).*

***Table S4. Self-reported vection analysis.***

|  | F value | Probability | $\eta_{p}^{2}$ |
| --- | --- | --- | --- |
| Buoyancy | F(1,22)=0.599 | 0.447 | 0.027 |
| Posture | F(1,22)=1.868 | 0.186 | 0.078 |
| Buoyancy x Posture | F(1,22)=1.868 | 0.186 | 0.078 |

***Table S5. Perception of size analysis.***

|  | F value | Probability | $\eta_{p}^{2}$ |
| --- | --- | --- | --- |
| Posture | F(1,21)=1.419 | 0.247 | 0.063 |
| Buoyancy | F(1,21)=0.143 | 0.709 | 0.007 |
| Distance | F(1.226,25.749)=3.235 | 0.076 | 0.133 |
| Posture x Buoyancy | F(1,21)=0.001 | 0.998 | 0.001 |
| Posture x Distance | F(2,42)=0.795 | 0.458 | 0.036 |
| Buoyancy x Distance | F(1.455,30.561)=0.822 | 0.414 | 0.038 |
| Distance x Buoyancy x Posture | F(2,42)=0.156 | 0.856 | 0.007 |

*
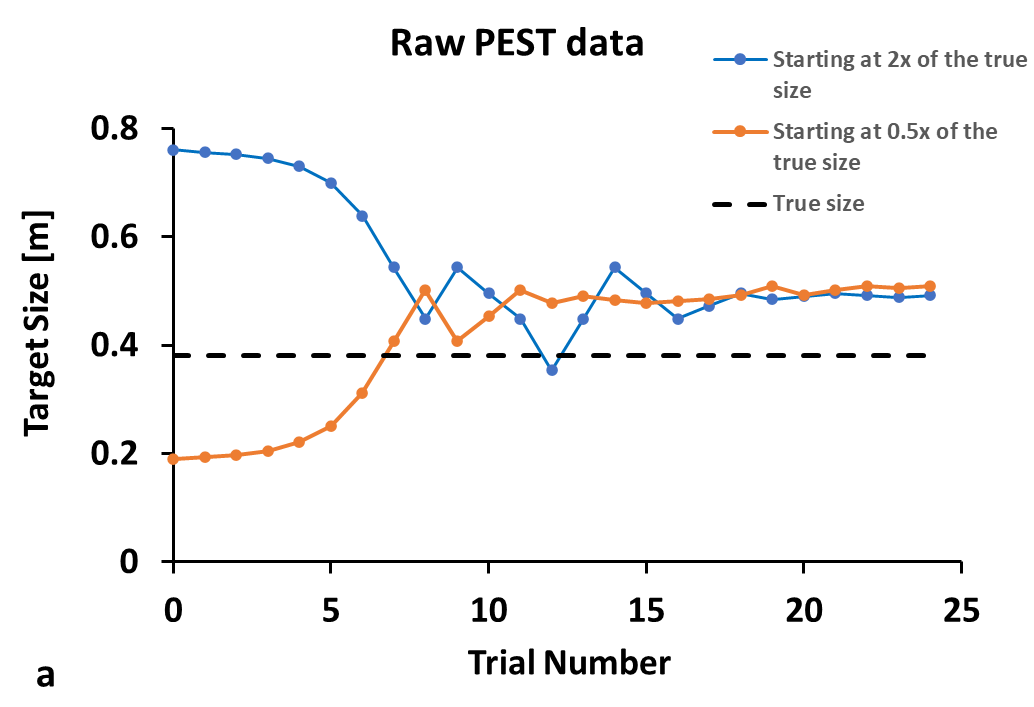

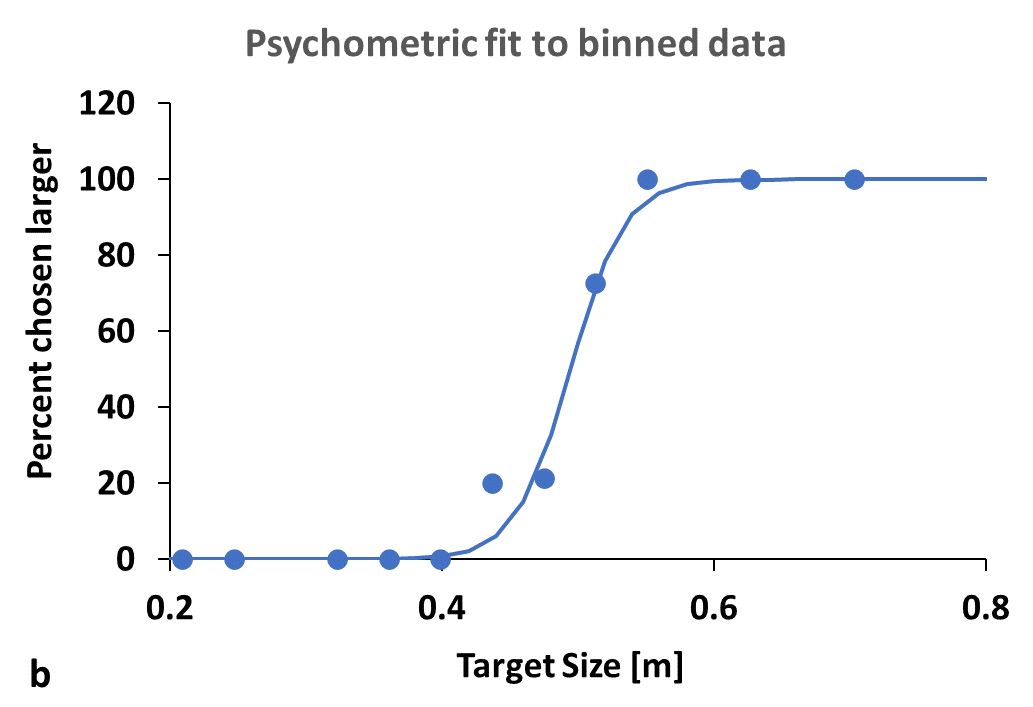
*

***Figure S3. Size/Distance Perception task results for a single target distance.*** *(a) shows the progression of the two pests, one initialized at one extreme, the other at the other. Over time these two tests collapse to a common value. (b) shows the process of integrating the results. Sample target size responses (-1 too small, +1 too large) binned and averaged. These values are then fit with a psychometric function (sold line)* *and the 50% point extracted. The initial extremes for the probe are set at 0.5 and 2 times the true stick length (38.1cm).*
